# Multiple Sclerosis in a Multi-Ethnic Population in Houston, Texas: A Retrospective Analysis

**DOI:** 10.3390/biomedicines8120534

**Published:** 2020-11-25

**Authors:** Vicki Mercado, Deepa Dongarwar, Kristen Fisher, Hamisu M. Salihu, George J. Hutton, Fernando X. Cuascut

**Affiliations:** 1 Immunology and Microbiology Graduate Program, Baylor College of Medicine, Houston, TX 77030, USA; Vicki.Mercado@bcm.edu; 2 Medical Scientist Training Program, Baylor College of Medicine, Houston, TX 77030, USA; 3 Center of Excellence in Health Equity, Training and Research Program, Baylor College of Medicine, Houston, TX 77030, USA; deepa.dongarwar@bcm.edu; 4 Texas Children Hospital, Blue Bird Circle Clinic for Multiple Sclerosis, Houston, TX 77030, USA; Kristen.Fisher@bcm.edu; 5 Department of Family & Community Medicine, Baylor College of Medicine, Houston, TX 77030, USA; hamisu.salihu@bcm.edu; 6 Baylor College of Medicine, Maxine Mesinger Multiple Sclerosis Center, Houston, TX 77030, USA; ghutton@bcm.edu

**Keywords:** multiple sclerosis, MS, disparities, minority populations

## Abstract

Multiple Sclerosis (MS) is a progressive neurodegenerative disease that affects more than 2 million people worldwide. Increasing knowledge about MS in different populations has advanced our understanding of disease epidemiology and variation in the natural history of MS among White and minority populations. In addition to differences in incidence, African American (AA) and Hispanic patients have greater disease burden and disability in earlier stages of disease compared to White patients. To further characterize MS in AA and Hispanic populations, we conducted a retrospective chart analysis of 112 patients treated at an MS center in Houston, Texas. Here, we describe similarities and differences in clinical presentation, MRI findings, treatment regimens, disability progression, and relapse rate. While we found several similarities between the groups regarding mean age, disability severity, and degree of brain atrophy at diagnosis, we also describe a few divergences. Interestingly, we found that patients who were evaluated by a neurologist at symptom onset had significantly decreased odds of greater disability [defined as Expanded Disability Status Scale (EDSS) > 4.5] at last presentation compared to patients who were not evaluated by a neurologist (OR: 0.04, 95% CI: 0.16–0.9). We also found that active smokers had significantly increased odds of greater disability both at diagnosis and at last clinical encounter compared to nonsmokers (OR: 2.44, 95% CI: 1.10–7.10, OR= 2.44, 95% CI: 1.35–6.12, *p* = 0.01, respectively). Additionally, we observed significant differences in treatment adherence between groups. Assessment of the degree of brain atrophy and progression over time, along with an enumeration of T1, T2, and gadolinium-enhancing brain lesions, did not reveal differences across groups.

## 1. Introduction

Multiple Sclerosis (MS) is an autoimmune inflammatory demyelinating condition that affects more than 2 million people worldwide [1,2]. A recent study estimates that in 2017, nearly 1 million adults had MS in the United States [1]. MS leads to an accumulation of disability over time, although disease-modifying therapies (DMT) may lessen long-term disability severity in most patients [3]. MS is considered a heterogeneous disease thought to result from a complex interaction among genetic predisposition, sex, and environment [4]. Increasing evidence suggests that racial disparities are important factors that may explain differences in the disease course, prevalence, incidence, and outcomes [5,6,7,8]. Despite comprising 13.4% and 18.3% of the American population, African-Americans (AA) and Hispanics, respectively, remain largely underrepresented and understudied in clinical trials [9,10,11]. Fortunately, an accumulating body of work characterizes MS in diverse populations. This development could improve our understanding of disease course and epidemiology and uncover disparities across various racial/ethnic groups. Better understanding disparities in MS clinical course and outcomes will allow for the development of more effective disease management in patients of diverse backgrounds.

Historically, it had been widely accepted that MS incidence was higher in the White population compared to the AA population [12]. However, population-based cohort studies have challenged this paradigm. A 2013 retrospective cohort study found that AA had a 47% increased risk of MS compared to Whites [13]. Disparities in MS clinical course in minority populations also encompass disability progression, disease burden, symptom presentation, and relapse rates. AA and Hispanics with MS have a higher disease burden and more severe disability in earlier stages of disease than White patients [10,14,15,16]. Additionally, AA patients commonly have multi-symptomatic presentation and early motor system involvement [14,17]. AA also experience inadequate recovery from symptoms and have shorter intervals between clinical attacks [7,8]. Furthermore, amongst MS individuals admitted to US nursing homes, AA patients are younger and more disabled than White patients [18]. Studies comparing MRI findings between AA and White patients revealed that the former show an increased degree of T2 hyperintense lesions and T1 hypointense lesions, which correlate with greater MS-related disability [19].

Clinical data for MS in the Hispanic population is comparatively limited. The few studies on Hispanics suggest a more rapid disability accumulation over time compared to White patients [20,21,22]. Interestingly, Hispanics were found to have a 50% decreased risk of developing MS compared to Whites [13]. However, several studies concur that Hispanics may have an earlier age of disease onset compared with other patient cohorts [13,20]. Hispanics and AA with MS are less likely than their White counterparts to visit a neurologist or MS specialist for disease management and have decreased rates of DMT usage due to noncompliance or inappropriate understanding of the treatment plan [23,24]. DMTs are critical for effective management and reduction of long-term disability in MS patients. In assessing these data, it is essential to consider that the Hispanic population is multiethnic and diverse. Other compounding factors that should be considered include socioeconomic status, place of birth, age of migration to the US, health literacy, systemic biases and systematic racism in healthcare, and access to care [5,20,25].

Much of our understanding of MS manifestation and clinical course in minority populations have come from a limited set of studies. Clinical trials on DMTs mostly lack data for minorities despite mounting evidence that these groups are at higher risk for a more aggressive disease course [26]. Approximately only 1% of the MS literature focuses on minority populations [10]. The purpose of this study was to address this lack of information by describing the clinical presentation, MRI findings, treatment regimens, disability progression, and relapse patterns in a racially and ethnically diverse population of MS patients in Houston, Texas. Given that the data for this study were collected from a clinic that predominantly serves patients of low socioeconomic status (SES), this study captures ethnic and racial disparities in MS among patients with a similar SES, potentially decreasing the possible effects of confounding factors. This study is critical and timely because it adds to an emerging literature that explores disparity in MS disease progression in AA and Hispanic MS patients compared to their White counterparts.

## 2. Patients and Methods

### 2.1. Study Design and Setting

Subjects were identified by a retrospective chart review of patients treated at the Smith Clinic Multiple Sclerosis Center. Smith clinic is a unique center that is part of a network that specifically cares for underserved and low socio-economic groups in Harris County, which includes the city of Houston. Additionally, Harris County is the third most populous county in the US. The majority of the patient population seen in the clinic are Non-Hispanic Black (NH-Black) or of Hispanic descent, and Mexicans constitute the majority of the Hispanic population served at the clinic. There is also a small percentage of Non-Hispanic White (NH-White) patients seen in the clinic. For the purposes of this study we are using the terms NH-Black and NH-White to account for the racial diversity of Hispanics seen in our clinic. Patients are attended to irrespective of insurance status or ability to pay.

### 2.2. Cohort Identification and Selection

Information from all patients who visited Smith clinic from March 2019 to March 2020 was identified through chart review and included in this retrospective study. All patients with a diagnosis of Relapsing Remitting MS (RRMS), Secondary Progressive MS (SPMS), or Primary Progressive MS (PPMS) were included.

### 2.3. Outcome Measurements

The following pre-selected information was abstracted for each patient: year and age of first symptoms, age at diagnosis, the amount of time that elapsed between onset of symptoms and diagnosis, disease subtype, estimated Expanded Disability Status Scale (EDSS) at diagnosis and last encounter, Disease Modifying Therapy (DMT) history (adverse reactions, relapses, and changes in immunomodulatory therapy), radiological findings, number of clinical relapses, smoking status, and autoimmune comorbidities. Escalation therapies included Glatiramer Acetate, Interferons, Teriflunomide, Dimethyl Fumarate and Fingolimod. High efficacy therapies included Rituximab, Ocrelizumab, Alemtuzumab and Natalizumab. Symptoms at disease onset were recorded and included motor, sensory, cerebellar, brainstem, bowel, and bladder function among others.

### 2.4. Data Collection and Management

Two neurologists extracted patient data from medical records and the study protocol was approved by an Institutional Review Board. Information from the most recent clinical encounter and from the clinical encounter at diagnosis was included. The EDSS at presentation was estimated based on the first documented neurologic examination by a neurologist and was not indicative of the maximal neurologic deficit during the demyelinating episode that led to the diagnosis. Severe disability was defined as an estimated EDSS score > 4.5. MRI interpretations were collected from radiology reports. Lesion quantification and atrophy scoring were extracted directly from radiology reports and raw images were not independently interpreted by the neurologists gathering the data. A relapse was defined as a new, documented, neurological complaint lasting more than 24 h with objective findings in the documented neurological exam, or a follow-up MRI showing new enhancing lesions.

### 2.5. Statistical Analysis

The statistical analyses were performed using R (version 3∙6∙1, Vienna, Austria) and RStudio (Version 1∙2∙5001, Boston, MA, USA). Based on the race and ethnicity information of the patients, we created a composite variable called ‘race/ethnicity’ and categorized the responses as Non-Hispanic (NH) White, NH-Black, Hispanic and ‘others’. We conducted descriptive statistics on patient socio-demographic and disease characteristics stratified by race/ethnicity. We conducted Fisher’s exact tests (for categorical variables) and ANOVA (for continuous variables). We examined the usage and impact of DMTs across racial/ethnic groups. We also examined various markers of disease progression including lesions and atrophy in the brain as well as the thoracic and cervical spine stratified by race/ethnicity using Fisher’s exact test. Applying adjusted Exact logistic regression models, we evaluated the association between various patient characteristics and a high EDSS score (EDSS > 4.5). Models were adjusted for different covariates based on the literature and context, along with experts’ recommendations. All analyses were based on two-tailed probabilities with a type 1 error rate set at 5%.

## 3. Results

Data from a total of 114 patients were analyzed in this study. Two patients were excluded due to a substantial amount of missing information. Of the included 112 patients, most were diagnosed with Relapsing Remitting MS (RRMS). About 73% of NH-White, 92% of NH-Black, and 95% of Hispanic patients had RRMS, whereas only 18% of NH-White, 5% of NH-Black, and 2.5% of Hispanic patients were diagnosed with Primary Progressive MS (PPMS) (Table 1). One Hispanic patient had a diagnosis of SPMS. There were no significant differences among the groups with regard to MS type at diagnosis (*p* = 0.1859), or smoking status (*p* = 0.3079). All groups had a similar female to male ratio, with a greater proportion of female MS patients (Table 1, *p* = 0.3675). Average age at diagnosis (*p* = 0.9918) and mean time to diagnosis (*p* = 0.9934) were also similar across all groups (Table 1). Interestingly, between the groups, we found significant differences in the percentage of patients who were adherent or experienced relapse while on escalation or high efficacy therapies. Specifically, 63.2% of NH-White, 73% of NH-Black, and 61.8% of Hispanic patients were adherent to escalation therapy (Table 1, *p* = 0.0252). 100% of NH-White, 84.2% of NH-Black, and 50% of Hispanic patients were adherent to high efficacy therapy (Table 1, *p* = 0.0252). 26.3% of NH-White, 31.1% of NH-Black, and 36.4% of Hispanic patients relapsed while on escalation therapy (Table 1, *p* = 0.000151). 0% of NH-White, 10.5% of NH-Black, and 0% of Hispanic patients relapsed while on high efficacy therapy (Table 1, *p* = 0.00015). Of note, one of the reasons for relapse includes non-adherence; thus interpretation of relapse data must consider the adherence percentages presented.

Notably, only 28% of the NH-Black population had received an evaluation by a neurologist at symptom onset, whereas 53% of Hispanic and 45% of NH-White patients had, although this was not statistically significant (Table 1, *p* = 0.1778). In this cohort, there were no statistically significant differences in receipt of a medical evaluation at symptom onset; 63–70% of patients from all groups were able to access medical evaluation. Additionally, NH-White, NH-Black and Hispanic patients exhibited no differences in symptoms at diagnosis or mean EDSS score at diagnosis and last encounter (Table 2). There was a significant difference in the percentage of patients with severe disability (EDSS score > 4.5) at diagnosis and at last encounter; 14.3% of NH-White MS patients had severe disability at diagnosis compared to 50% of NH-Black and 31.6% of Hispanic patients (Table 2, *p* < 0.001). This was also true at last encounter with 32.5% of NH-White, 45.5% of NH-Black and 41% of Hispanic MS patients with severe disability at their most recent clinical visit (Table 2, *p* < 0.001).

**Figure 1:**
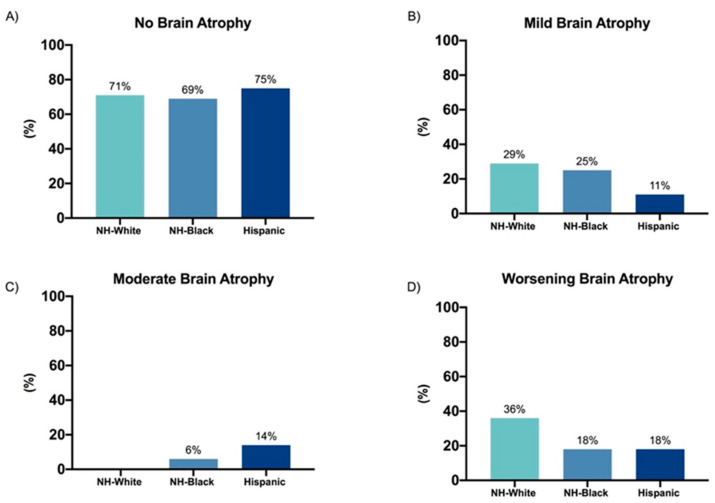
Degree of brain atrophy at diagnosis and worsening of brain atrophy from diagnosis to most recent MRI scan. Presented as the total percentage of each group, is the proportion of patients who had none (**A**), mild (**B**), or moderate (**C**) brain atrophy at the time of diagnosis, as well as the proportion of patients who had increased brain atrophy in their most recent MRI scan compared to diagnosis (**D**). Only patients who had MRI scans on file were included in this analysis. *p* = 0.5155 for comparison between degree of brain atrophy (none, mild, moderate) (Fisher’s exact). *p* = 0.3387 for comparison of total percentage of patients who had worsening brain atrophy on most recent MRI compared to diagnosis (Fisher’s exact).

**Figure 2:**
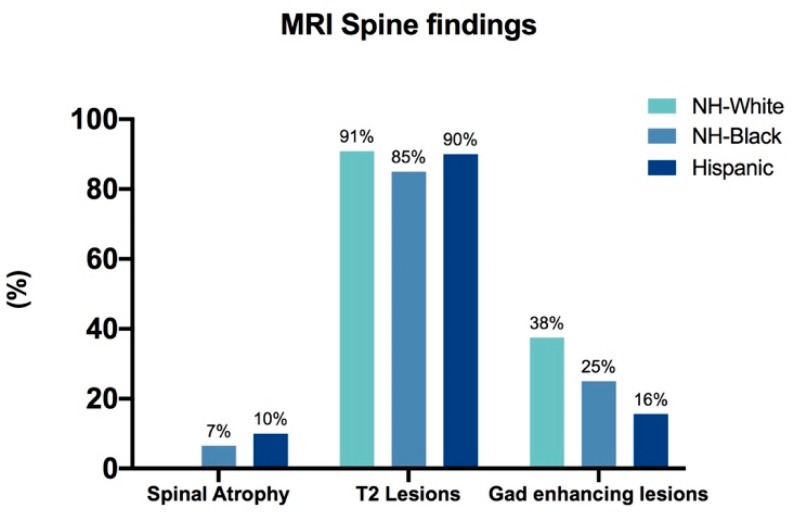
The total percentage of patients in each group who had spinal atrophy, T2, or gadolinium-enhancing lesions in the spine as determined by MRI findings at diagnosis. Only patients who had MRI scans on file were included in this analysis. *p* = 0.6974, *p* = 0.5128, *p* = 0.2957 for comparison of total percentage of patients in each group that had spinal atrophy, spinal T2 lesions, and spinal gadolinium-enhancing lesions respectively (Fisher’s exact).

Assessment of degree of brain atrophy and progression over time revealed that NH-White, NH-Black and Hispanic patients in this cohort had a similar degree of brain atrophy at diagnosis and over time (Figure 1). Enumeration of T1, T2, and gadolinium-enhancing brain lesions at diagnosis also showed no significant differences between the groups (data not shown). Spinal atrophy and quantity of T2 and gadolinium-enhancing lesions in the spine at diagnosis and at last presentation were also similar between groups (Figure 2).

Patient usage of escalation or high efficacy therapies did not significantly impact the patient’s likelihood of having an EDSS score > 4.5 at last clinical encounter after adjustment for adherence, smoking, race, age, prior exposure to escalation therapies, and EDSS at diagnosis (Table 3). Active smokers were 2.44 times as likely to have an EDSS score > 4.5 at their last clinical encounter compared to non-smokers after adjustment for age and race (OR: 2.44, 95% CI: 1.36–6.12, *p* = 0.01) (Table 3). Interestingly, after adjustment for race and age, patients who were evaluated by a neurologist at diagnosis had significantly lower adjusted odds of an EDSS score > 4.5 at last presentation compared to patients who were not evaluated by a neurologist (OR: 0.40, 95% CI: 0.16–0.90, *p* = 0.04) (Table 3).

Active smokers were 2.79 times as likely to have an EDSS score > 4.5 at diagnosis compared to non-smokers after adjustment for age and race (OR: 2.79, 95% CI: 1.10–7.10, *p* = 0.01) (Table 4). There was no significant association between time to diagnosis and having a high EDSS score at diagnosis (Table 4). There were no significant differences in total relapse occurrence for patients on escalation therapy vs. high efficacy therapy for each racial/ethnic group (data not shown). Of 24 NH-white patients, 19 had ever used escalation therapy, and 5 had used high efficacy therapy. Of 93 NH-Black patients, 74 had used escalation therapy, and 19 had used high efficacy therapy. For the Hispanic patients group of 63 patients, 55 had ever used escalation therapy while 18 had documented high efficacy therapy use. We found no differences between the groups concerning the usage of escalation vs. high efficacy therapies.

## 4. Discussion

The goal of this retrospective cohort study was to describe MS patient characteristics in a multi-ethnic population in Houston and compare findings between racial/ethnic groups. Our study demonstrates several racial/ethnic similarities and a few differences in multiple sclerosis presentation and disease course. We found that the groups had a similar mean age at diagnosis, mean EDSS score at diagnosis and last presentation, and a similar degree of brain and spinal atrophy at diagnosis as well. MRI spinal findings were also comparable between NH-White, Black and Hispanic groups. The average time from symptom onset to diagnosis, and overall symptom presentation, were also similar between the groups. The clinic that these patients were treated at is a hub for the underserved and low socioeconomic communities. Thus, we suspect that many of the patients in this cohort were of a similar socioeconomic background, which undoubtedly can influence disease manifestation and outcomes. It is plausible that these similar environmental factors, along with the small sample size may explain the many similarities detected between the groups. However, further studies are required to evaluate this hypothesis.

Interestingly, after adjustment for race and age, patients who were evaluated by a neurologist at diagnosis had 60% lower odds (OR = 0.40, 95% CI: 0.16–0.90) of an EDSS score > 4.5 at last presentation compared to patients who were evaluated by a non-neurology specialist. This suggests a logical protective effect of treatment by a neurologist at symptom onset and highlights the importance of access to treatment for all patients. Indeed, a national descriptive study found that people with MS who saw a neurologist were more likely to receive appropriate DMT treatment and see rehabilitation and urologist specialists compared to people who saw other providers [27]. A 2017 study on racial disparities in neurologic health care access revealed that Black patients were 30% less likely to see an outpatient neurologist and were more likely to be cared for in the emergency department compared to their White counterparts [23]. Similarly, Hispanic patients were 40% less likely to see an outpatient neurologist compared to NH-Whites [23].

We found that actively smoking patients were 2.44 times as likely (95% CI: 1.36–6.12) to have severe disability at diagnosis and at the last clinic follow up. A recent systematic review and meta-analysis found evidence supporting the causal involvement of smoking in the development and progression of MS [28]. Altogether, these data suggest that smoking prevention and cessation education programs and early intervention by a neurologist should be implemented to achieve optimal MS care in diverse patient populations.

Consistent with published reports, a greater proportion of NH-Black patients had early severe disability (defined in our study as an estimated EDSS score > 4.5) when compared to NH-White and Hispanic patients [29,30]. In our present study, treatment modality did not impact the risk of having an estimated EDSS score > 4.5 at the last visit. Nonetheless, we observed a trend towards a higher relapse rate in escalation therapies vs. high efficacy therapies, especially in NH-Blacks. We also observed significant differences in adherence between the groups. Interestingly, a greater percentage of NH-Black patients relapsed while on high efficacy therapy compared to Hispanic patients, despite having greater adherence. Other studies have found that NH-Black patients treated with interferons experienced more relapses and new MS lesions on T2-weighted brain magnetic imaging than NH-Whites [31]. However, further studies on the interaction between race/ethnicity and DMT response for MS are necessary.

Several studies have shown that African Americans have significantly higher CNS lesion burden, more frequent relapses, worse ambulatory disability, worse post-relapse recoveries, and higher overall disability at diagnosis [5,10,19,29]. Overall, our findings did not confirm these prior observations and we believe that the similar socioeconomic background of this patient cohort, along with the small sample size, may have contributed to this. Nevertheless, it is evident that further studies are needed to investigate the various environmental and social factors contributing to divergent MS clinical course outcomes between diverse populations. 

Limitations of this study include its retrospective nature, the variable periods of follow-up and the selection of therapy by the treating physician (nonrandomized). The study was also constrained by a small sample size, which could have induced a type 2 error leading to the inability to reject the null hypothesis in some of our comparisons. Additionally, our interpretation of the relapse data is limited because one of the possible reasons for relapse is non-adherence. Thus, relapse data are not corrected for the degree of non-adherence and should be assessed accordingly. Lastly, it is important to note that we did not analyze the imaging data ourselves. Instead, we collected information from MRI reports. Often, the number of lesions was documented as a range, thereby limiting data precision. Moreover, the atrophy measurements were subjective rather than objective quantification, and some patients were missing MRI information at diagnosis (e.g., performed at a different institution). These limitations may have impacted the capability to show radiological differences at presentation between groups.

Our study is important because it adds to emerging literature describing disease characteristics in minority populations with MS. The disparities in MS progression, onset, and disease course warrants further study. Of 60,000 published articles on MS, only 113 focused on NH-Black and only 23 focused on Hispanic American patients with MS as of 2014 [10]. This demonstrates a need for studies that are intentionally inclusive of these populations. Since 2014, there has been a modest but steady increase in studies focused on these populations. There is a clear disparity in MS treatment access for patients from different racial and ethnic backgrounds. Drivers of disparity are often comprised of complex interactions among factors such as socioeconomic status, access to healthcare and wellness resources (clinics, hospitals, grocery stores, fitness centers), systemic racism and biases in healthcare, and limited health literacy. This systemic web of disparity can be challenging to disentangle, but understanding it is necessary for improving the care of minority patients with MS.

Future prospective randomized controlled trials in different racial/ethnic groups with MS are essential to better understand the disease progression, management and treatment outcomes for diverse patient populations.

## Figures and Tables

**Table 1:** Diagnosis Characteristics of patients with MS stratified by race/ethnicity.

Characteristics	NH-White (*n* = 11)	NH-Black (*n* = 61)	Hispanic (*n* = 40)	*p* Value
**Multiple Sclerosis (MS) type at diagnosis**				*p* = 0.1859
Relapsing remitting MS	82%	95%	95%	
Primary progressive MS	18%	5%	2.5%	
Secondary progressive MS	0%	0%	2.5%	
**Mean Age at diagnosis (years)**	39.9 (11.3)	36.7 (11.4)	32.4 (11.5)	*p* = 0.9918
**Female/Male ratio**	1.70/1	2.33/1	1.22/1	*p* = 0.3765
**Active smokers**	55%	44%	30%	*p* = 0.3079
**Mean time from symptom onset to diagnosis (months)**	30.8 (38.9)	32.9 (32.1)	13.7 (15.4)	*p* = 0.9934
**Medical Evaluation at symptom onset**	64%	63%	70%	*p* = 0.8597
**Neurological Evaluation at symptom onset**	45%	28%	53%	*p* = 0.1778
**Adherence** (Adherence/Ever used)				*p* = 0.0252
Escalation therapy	63.2%	73%	61.8%	
High efficacy therapy	100%	84.2%	50%	
**Relapse** (Relapse/Ever used)				*p* = 0.00015
Escalation therapy	26.3%	31.1%	36.4%	
High efficacy therapy	0%	10.5%	0%	

EDSS score at diagnosis and EDSS score at last clinical visit were compared within each group. Standard deviation is shown in parentheses. *p* = 0.4253 (NH-Black), *p* = 0.1757 (Hispanic), *p* = 0.0324 (NH-White), (paired sample *t*-test). For adherence and relapse data, chi-squared test and Fisher’s-exact test were used respectively. Escalation therapies included Glatiramer Acetate, Interferons, Teriflunomide, Dimethyl Fumarate and Fingolimod. High efficacy therapies included Rituximab, Ocrelizumab, Alemtuzumab and Natalizumab.

**Table 2:** Clinical characteristics of MS patients by race/ethnicity.

Clinical Characteristics	NH-White (*n* = 11)	NH-Black (*n* = 61)	Hispanic (*n* = 40)	*p* Value
**EDSS scores**				
Mean EDSS score at diagnosis (SD)	2.6 (2.1)	2.2 (1.1)	3.8 (1.9)	*p* = 0.9328
Mean EDSS score at last presentation (SD)	2.9 (2.8)	4.2 (2.9)	3.8 (2.3)	*p* = 0.9950
Severe disability at diagnosis (EDSS > 4.5)	14.3%	50%	31.6%	*p* = < 0.001
Severe disability at last presentation (EDSS > 4.5)	32.5%	45.5%	41%	*p* = < 0.001
**Symptoms at Presentation**				*p* = 0.1473
Motor	72.7%	57.4%	47.5%	
Brainstem	27.3%	24.9%	25%	
Cerebellar	27.3%	37.7%	37.5%	
Gait	27.3%	26.2%	15%	
Sensory	72.7%	37.7%	52.5%	
Visual	9.1%	27.9%	30%	
Cognitive	9.1%	9.8%	5%	
Other or unknown	36.4%	18.1%	15%	

**Table 3:** Association between various patient characteristics and high EDSS score (>4.5) at last presentation.

High EDSS Score at Last Presentation		
	OR	*p*-Value
**Usage of escalation therapies ^a^**		
No	reference	
Yes	1.60 (0.45–6.14)	0.48
**Usage of high efficacy therapies ^b^**		
No	reference	
Yes	2.64 (0.87–8.33)	0.09
**Smoker ^c^**		
No	reference	
Yes	2.44 (1.36–6.12)	0.01
**Medical evaluation by Neurologist ^c^**		
No	reference	
Yes	0.40 (0.16–0.90)	0.04
**Adherence to DMT ^c^**		
Yes	reference	
No	0.73 (0.31–1.62)	0.43
**Time to diagnosis ^c^**		
<=12 months	reference	
>12 months	1.73 (0.75–4.01)	0.2

^a^ adjusted for adherence, smoking, race and age and EDSS at diagnosis; ^b^ adjusted for prior exposure to escalation therapies, adherence, smoking, race, age and EDSS at diagnosis; ^c^ adjusted for age and race.

**Table 4:** Association between various patient characteristics and high EDSS score at diagnosis (>4.5).

High EDSS Score at Diagnosis		
	OR	*p*-Value
**Smoker ^c^**		
No	reference	
Yes	2.79 (1.10–7.10)	0.01
**Time to diagnosis ^c^**		
<=12 months	reference	
>12 months	1.15 (0.46–2.83)	0.77

^c^ adjusted for age and race.
